# Identifying psychological and clinical risk factors for moderate-to-severe tinnitus in older patients with hearing loss: a multivariable prediction model

**DOI:** 10.3389/fneur.2025.1647071

**Published:** 2025-07-23

**Authors:** Chenguang Zhang, Yicong Wang, Chunlong Zhao, Rou Xue, Chenghao Hu, Bin Guo

**Affiliations:** ^1^Graduate School of Qinghai University, Xining, China; ^2^Department of Otolaryngology, Affiliated Hospital of Qinghai University, Xining, China; ^3^Department of Gastrointestinal Oncology, Affiliated Hospital of Qinghai University, Xining, China

**Keywords:** tinnitus, anxiety, poor sleep quality, hearing loss, nomogram

## Abstract

**Objective:**

To develop and validate a clinical prediction model for moderate-to-severe tinnitus (THI ≥ 38) in patients with hearing loss and to identify the key psychological and clinical factors associated with its risk.

**Methods:**

This retrospective single-centre study included 301 patients with hearing loss who visited Qinghai University Affiliated Hospital between August 2024 and May 2025. The cohort was randomly divided into a training set (*n* = 210) and a validation set (*n* = 91) in a 7:3 ratio. Moderate-to-severe tinnitus served as the outcome of interest. Psychological and clinical risk factors were initially screened using univariate logistic regression, and variables with *p* < 0.05 were subsequently included in a multivariable logistic regression model.

**Results:**

The final multivariable model identified five independent psychological and clinical risk factors for moderate-to-severe tinnitus: older age (OR = 2.415), hypertension (OR = 2.120), poor sleep quality (OR = 2.821), anxiety (OR = 1.967), and severe hearing loss (OR = 3.452). The model demonstrated good discriminative performance, with an AUC of 0.734 in the training set and 0.760 in the validation set.

**Conclusion:**

In patients with hearing loss, psychological and clinical risk factors—including poor sleep quality, anxiety, hypertension, and severe hearing loss—were significantly associated with moderate-to-severe tinnitus. These findings underscore the need for integrated management strategies that address both psychological and clinical components of tinnitus risk.

## 1 Introduction

Tinnitus is defined as the perception of sound in the absence of an external auditory stimulus. It is relatively common in adults, especially among the elderly. Recent global epidemiological data indicate that approximately 14% of adults experience tinnitus, with about 2% suffering from severe forms that significantly impair quality of life (1). The prevalence of tinnitus increases markedly with age, making it a common comorbidity in older populations (2). Among the various risk factors, hearing loss is widely recognized as the most important predictor of tinnitus (3–5). Chen et al. also suggested a potential association between laryngopharyngeal reflux (LPR) and tinnitus, highlighting possible shared pathophysiological mechanisms (6). In addition, tinnitus is increasingly viewed as a condition influenced by systemic and vascular factors. Studies have linked it to comorbidities such as dyslipidemia, atherosclerosis, thyroid dysfunction, and psychiatric disorders, as well as vascular and neurological abnormalities like carotid plaques and white matter lesions (7, 8).

Age-related hearing loss (ARHL), also known as presbycusis, is highly prevalent in older adults and is closely associated with both the presence and severity of tinnitus. Several studies have reported that individuals with hearing loss are two to three times more likely to report tinnitus compared to those with normal hearing (3, 4). Even after controlling for auditory thresholds, age itself remains an independent risk factor, suggesting that age-related mechanisms beyond the auditory system may contribute to tinnitus onset (2). Other factors such as vertigo, poor sleep quality, and chronic head or neck pain have also been shown to further increase tinnitus risk (9). Beyond auditory pathology, growing evidence indicates that psychological factors also play a significant role in the development and persistence of tinnitus (5, 10, 11). Depression and anxiety are among the most common psychiatric comorbidities in tinnitus patients. Systematic reviews have found that approximately one-third of individuals with tinnitus meet the criteria for clinically significant depression (11, 12). A large-scale cohort study by Oosterloo et al. demonstrated that even mild tinnitus was strongly associated with elevated levels of depressive and anxiety symptoms (13). In addition, certain psychological traits such as neuroticism and somatization may increase both the likelihood of tinnitus and the degree of subjective distress it causes (14). Given the multifactorial nature of tinnitus, recent research has focused on developing multivariable prediction models to enable early identification and intervention for high-risk individuals. Rademaker et al. developed a logistic regression model incorporating factors such as age, sleep quality, and hearing aid use, which successfully predicted tinnitus presence in the general population (15). Hobeika and Jafari further applied machine learning methods to large-scale biobank and clinical datasets, integrating psychological and clinical risk factors to construct high-accuracy models with good external validation performance (16, 17). These studies provide a theoretical foundation and practical tools for precision prediction and individualized intervention in tinnitus management. Despite these advances, prediction models specifically targeting older adults with hearing loss remain limited, particularly those that integrate both psychological and clinical risk factors. Therefore, this study aimed to identify key risk factors associated with tinnitus among individuals with hearing impairment, with a special focus on older adults. Based on these findings, we sought to develop a clinically applicable prediction model incorporating psychological and clinical risk factors to improve early recognition and promote personalized management of tinnitus in clinical practice.

## 2 Materials and methods

### 2.1 Patient selection

This was a single-centre, retrospective observational study conducted at Qinghai University Affiliated Hospital between August 2024 and May 2025. A total of 301 patients with hearing loss were included.

Inclusion criteria were as follows: (1) age ≥18 years; (2) completed audiological assessments, medical history, and psychological questionnaires; (3) completion of the Tinnitus Handicap Inventory (THI) for evaluating tinnitus severity; and (4) availability of all key clinical variables required for the analysis. Exclusion criteria included: (1) active acute ear diseases (e.g., otitis externa or media, sudden sensorineural hearing loss); (2) history of cranial trauma or otologic surgery; (3) diagnosed neurodegenerative or severe psychiatric disorders; and (4) incomplete or missing data.

All eligible patients were randomly divided into a training set (*n* = 210) and a validation set (*n* = 91) in a 7:3 ratio. The study was approved by the Ethics Committee of Qinghai University Affiliated Hospital (Approval No. P-SL-2024-056), and written informed consent was obtained from all participants.

### 2.2 Variable selection and definitions

A total of 18 candidate variables were included in the analysis to develop the predictive model. The primary outcome was moderate-to-severe tinnitus in patients with hearing loss. According to the THI, tinnitus severity is categorized as follows: slight (0–16), mild (18–36), moderate (38–56), severe (58–76), and catastrophic (78–100) (18, 19). In this study, patients with a THI score ≥ 38—corresponding to moderate, severe, or catastrophic tinnitus—were classified as having moderate-to-severe tinnitus and coded as 1. Those with a THI score < 38 (i.e., slight or mild tinnitus), or with no tinnitus symptoms, were coded as 0. This binary classification was used as the outcome variable in model construction.

The predictors encompassed five domains: demographic, otological, clinical, psychological, and sleep-related factors. Demographic variables included age, gender, and BMI, which was categorized as underweight (<18.5), normal (18.5–23.9), overweight (24.0–27.9), or obese (≥28.0) based on Chinese criteria (20). Otological indicators included hearing loss severity—classified as mild (26–40 dB), moderate (41–60 dB), or severe (61–80 dB)—as well as the duration of hearing loss (<12 vs. ≥12 months), laterality (unilateral vs. bilateral), family history of hearing loss or tinnitus, history of ototoxic drug exposure, and occupational noise exposure (≥85 dB for ≥8 h/day for ≥1 year).

Clinical comorbidities included hypertension, diabetes, hyperlipidemia, and laryngopharyngeal reflux (LPR), the latter defined by a Reflux Symptom Index (RSI) ≥ 13 and a Reflux Finding Score (RFS) ≥ 7 (21, 22). Psychological variables included anxiety and depression, assessed using the Zung Self-Rating Anxiety Scale (SAS) and the Patient Health Questionnaire-9 (PHQ-9), respectively. Anxiety was defined as SAS ≥ 50, and depression as PHQ-9 ≥ 10 (23, 24). Sleep quality was assessed using the Pittsburgh Sleep Quality Index (PSQI), with a score >5 indicating poor sleep quality (25).

These variables were selected based on prior literature and clinical relevance, and were included in subsequent univariate and multivariable logistic regression analyses to construct the final prediction model.

### 2.3 Statistical analysis

All statistical analyses were conducted using R software (version 4.4.2). Continuous variables were tested for normality. Those with a normal distribution were presented as mean ± standard deviation and compared using the independent samples *t*-test, while non-normally distributed variables were expressed as median and interquartile range (IQR) and compared using the Mann–Whitney U test. Categorical variables were summarized as frequencies and percentages and compared using the chi-square test.

To identify predictors of moderate-to-severe tinnitus, univariate logistic regression analysis was first performed. Variables with a *p*-value < 0.05 in univariate analysis were subsequently included in a multivariate logistic regression model to identify independent risk factors. A predictive nomogram was constructed based on the final multivariable model.

Model performance was evaluated using several metrics: discrimination was assessed by the area under the receiver operating characteristic (ROC) curve (AUC) and concordance index (C-index); calibration was assessed via Brier score and calibration curves; and clinical utility was evaluated using decision curve analysis (DCA). Internal validation was performed using 1,000 bootstrap resamples. The Hosmer–Lemeshow goodness-of-fit test was used to assess model calibration. A two-sided *p*-value < 0.05 was considered statistically significant. All analyses and visualizations were implemented using R packages including rms, pROC, and rmda.

## 3 Result

### 3.1 Baseline characteristics

There were no statistically significant differences between the moderate-to-severe tinnitus group (THI ≥ 38, *n* = 186) and the no or slight-to-mild tinnitus group (THI < 38, *n* = 115), based on chi-square tests, in terms of gender (*p* = 0.702), diabetes (*p* = 0.283), smoking (*p* = 0.206), alcohol consumption (*p* = 0.471), duration of hearing loss ≥12 months (*p* = 0.778), history of LPR (*p* = 0.152), ototoxic drug use (*p* = 0.234), noise exposure (*p* = 0.081), family history (*p* = 0.226), hyperlipidemia (*p* = 0.896), or BMI category (*p* = 0.104).

In contrast, patients with moderate-to-severe tinnitus were significantly more likely to be of older age (≥60 years) (78.5% vs. 60.9%, *p* = 0.002), have hypertension (45.2% vs. 27.8%, *p* = 0.004), poor sleep quality (66.7% vs. 49.6%, *p* = 0.005), anxiety symptoms (57.5% vs. 40.9%, *p* = 0.007), depressive symptoms (55.4% vs. 35.7%, *p* = 0.001), severe hearing loss (39.8% vs. 22.6%, *p* < 0.001), and bilateral hearing loss (59.1% vs. 44.3%, *p* = 0.017).

Detailed comparisons of basic patient characteristics between the two groups are summarized in Table 1.

**Table 1:** Basic patient characteristics.

Variables	Total (*n* = 301)	Moderate-to-severe tinnitus (THI ≥38, *n* = 186)	No or mild tinnitus (THI <38, *n* = 115)	*p*-value
Age				0.002*
<60	85 (28.2%)	40 (21.5%)	45 (39.1%)	
≥60	216 (71.8%)	146 (78.5%)	70 (60.9%)	
Gender				0.702
Male	162 (53.8%)	98 (52.7%)	64 (55.7%)	
Female	139 (46.2%)	88 (47.3%)	51 (44.3%)	
Diabetes				0.283
No	206 (68.4%)	132 (71.0%)	74 (64.3%)	
Yes	95 (31.6%)	54 (29.0%)	41 (35.7%)	
Hypertension				0.004*
No	185 (61.5%)	102 (54.8%)	83 (72.2%)	
Yes	116 (38.5%)	84 (45.2%)	32 (27.8%)	
Smoking				0.206
No	184 (61.1%)	108 (58.1%)	76 (66.1%)	
Yes	117 (38.9%)	78 (41.9%)	39 (33.9%)	
Alcohol consumption				0.471
No	114 (37.9%)	67 (36.0%)	47 (40.9%)	
Yes	187 (62.1%)	119 (64.0%)	68 (59.1%)	
Poor sleep quality				0.005*
No	120 (39.9%)	62 (33.3%)	58 (50.4%)	
Yes	181 (60.1%)	124 (66.7%)	57 (49.6%)	
Anxiety				0.007*
No	147 (48.8%)	79 (42.5%)	68 (59.1%)	
Yes	154 (51.2%)	107 (57.5%)	47 (40.9%)	
Hearing loss severity				<0.001*
Mild	125 (41.5%)	59 (31.7%)	66 (57.4%)	
Moderate	76 (25.2%)	53 (28.5%)	23 (20.0%)	
Severe	100 (33.2%)	74 (39.8%)	26 (22.6%)	
Duration of hearing loss				0.778
<12 months	151 (50.2%)	95 (51.1%)	56 (48.7%)	
≥12 months	150 (49.8%)	91 (48.9%)	59 (51.3%)	
LPR				0.152
No	256 (85.0%)	163 (87.6%)	93 (80.9%)	
Yes	45 (15.0%)	23 (12.4%)	22 (19.1%)	
Hearing loss side				0.017*
Bilateral	161 (53.5%)	110 (59.1%)	51 (44.3%)	
Unilateral	140 (46.5%)	76 (40.9%)	64 (55.7%)	
Depression				0.001*
No	157 (52.2%)	83 (44.6%)	74 (64.3%)	
Yes	144 (47.8%)	103 (55.4%)	41 (35.7%)	
Ototoxic drug use				0.234
No	220 (73.1%)	131 (70.4%)	89 (77.4%)	
Yes	81 (26.9%)	55 (29.6%)	26 (22.6%)	
Noise exposure				0.081
No	217 (72.1%)	127 (68.3%)	90 (78.3%)	
Yes	84 (27.9%)	59 (31.7%)	25 (21.7%)	
Family history				0.226
No	287 (95.3%)	180 (96.8%)	107 (93.0%)	
Yes	14 (4.7%)	6 (3.2%)	8 (7.0%)	
Hyperlipidemia				0.896
No	212 (70.4%)	130 (69.9%)	82 (71.3%)	
Yes	89 (29.6%)	56 (30.1%)	33 (28.7%)	
BMI				0.104
Normal	156 (51.8%)	92 (49.5%)	64 (55.7%)	
Overweight	78 (25.9%)	44 (23.7%)	34 (29.6%)	
Obese	51 (16.9%)	38 (20.4%)	13 (11.3%)	
Underweight	16 (5.3%)	12 (6.5%)	4 (3.5%)	

BMI, body mass index; LPR, laryngopharyngeal reflux; THI, Tinnitus Handicap Inventory; Variables with *p*-value <0.05 were considered statistically significant and are marked with *.

### 3.2 Univariate and multivariable logistic regression analysis

Univariate logistic regression analysis was performed on all candidate variables in the training set. The results indicated that age, hypertension, poor sleep quality, anxiety, depression, noise exposure history, and hearing loss severity were significantly associated with the presence of moderate-to-severe tinnitus among patients with hearing loss (all *p* < 0.05). These significant variables were subsequently entered into a multivariable logistic regression model. The analysis revealed that age (OR = 2.415, 95% CI: 1.243–4.746, *p* = 0.010), hypertension (OR = 2.120, 95% CI: 1.113–4.134, *p* = 0.024), poor sleep quality (OR = 2.821, 95% CI: 1.477–5.533, *p* = 0.002), anxiety (OR = 1.967, 95% CI: 1.044–3.771, *p* = 0.038), and severe hearing loss (OR = 3.452, 95% CI: 1.629–7.631, *p* = 0.002) were independent predictors of moderate-to-severe tinnitus. Details of the univariate and multivariable analyses are presented in Tables 2, 3. These five variables were incorporated into the final prediction model and visualized using a nomogram.

**Table 2:** Univariate logistic regression analysis of risk factors for moderate-to-severe tinnitus in patients with hearing loss.

Variables	OR	95%CI	*p*-value
Age			
<60		Reference	
≥60	2.543	(1.377, 4.740)	0.003*
Gender			
Male		Reference	
Female	1.312	(0.750, 2.309)	0.343
Diabetes			
No		Reference	
Yes	0.858	(0.471, 1.574)	0.617
Hypertension			
No		Reference	
Yes	2.000	(1.118, 3.647)	0.021*
Smoking			
No		Reference	
Yes	1.147	(0.649, 2.044)	0.638
Alcohol consumption			
No		Reference	
Yes	1.177	(0.662, 2.087)	0.576
Poor sleep quality			
No		Reference	
Yes	2.023	(1.146, 3.595)	0.015*
Anxiety			
No		Reference	
Yes	1.768	(1.010, 3.124)	0.048*
Hearing loss severity			
Mild		Reference	
Moderate	2.167	(1.089, 4.418)	0.030*
Severe	2.611	(1.331, 5.268)	0.006*
Duration of hearing loss			
<12 months		Reference	
≥12 months	0.867	(0.496, 1.515)	0.617
LPR			
No		Reference	
Yes	0.602	(0.284, 1.280)	0.183
Hearing loss side			
Bilateral		Reference	
Unilateral	0.574	(0.326, 1.005)	0.053
Depression			
No		Reference	
Yes	2.135	(1.213, 3.803)	0.009*
Ototoxic drug use			
No		Reference	
Yes	1.588	(0.836, 3.112)	0.166
Noise exposure			
No		Reference	
Yes	1.996	(1.037, 4.007)	0.044*
Family history			
No		Reference	
Yes	0.600	(0.162, 2.222)	0.431
Hyperlipidemia			
No		Reference	
Yes	1.249	(0.675, 2.353)	0.484
BMI			
Normal		Reference	
Overweight	0.806	(0.408, 1.597)	0.534
Obese	1.773	(0.801, 4.179)	0.171
Underweight	1.478	(0.451, 5.723)	0.536

OR, odds ratio; CI, confidence interval; BMI, body mass index; LPR, laryngopharyngeal reflux; Variables with *p*-value <0.05 were considered statistically significant and are marked with *.

**Table 3:** Multivariable logistic regression analysis of risk factors for moderate-to-severe tinnitus in patients with hearing loss.

Variables	OR	95%CI	*p*-value
Age			
<60			
≥60	2.415	(1.243, 4.746)	0.010*
Hypertension			
No			
Yes	2.120	(1.113, 4.134)	0.024*
Poor sleep quality			
No			
Yes	2.821	(1.477, 5.533)	0.002*
Anxiety			
No			
Yes	1.967	(1.044, 3.771)	0.038*
Depression			
No			
Yes	1.789	(0.956, 3.374)	0.070
Noise exposure			
No			
Yes	2.057	(0.991, 4.452)	0.059
Hearing loss severity			
Mild			
Moderate	2.144	(1.000, 4.714)	0.053
Severe	3.452	(1.629, 7.631)	0.002*

OR, odds ratio; CI, confidence interval; Variables with *p*-value <0.05 were considered statistically significant and are marked with *.

### 3.3 Model construction

The predictive model was constructed based on the training set. Variables that were statistically significant in the univariate analysis (*p* < 0.05) were entered into a multivariable logistic regression model. Ultimately, five independent predictors of moderate-to-severe tinnitus were identified: age, hearing loss severity, poor sleep quality, anxiety, and hypertension (all *p* < 0.05). These variables formed the basis of the final prediction model. A nomogram was subsequently developed to provide individualized risk estimates for moderate-to-severe tinnitus in patients with hearing loss (Supplementary Figure 1), serving as a practical and visual tool for clinical risk assessment.

### 3.4 Model validation

**Figure 1:**
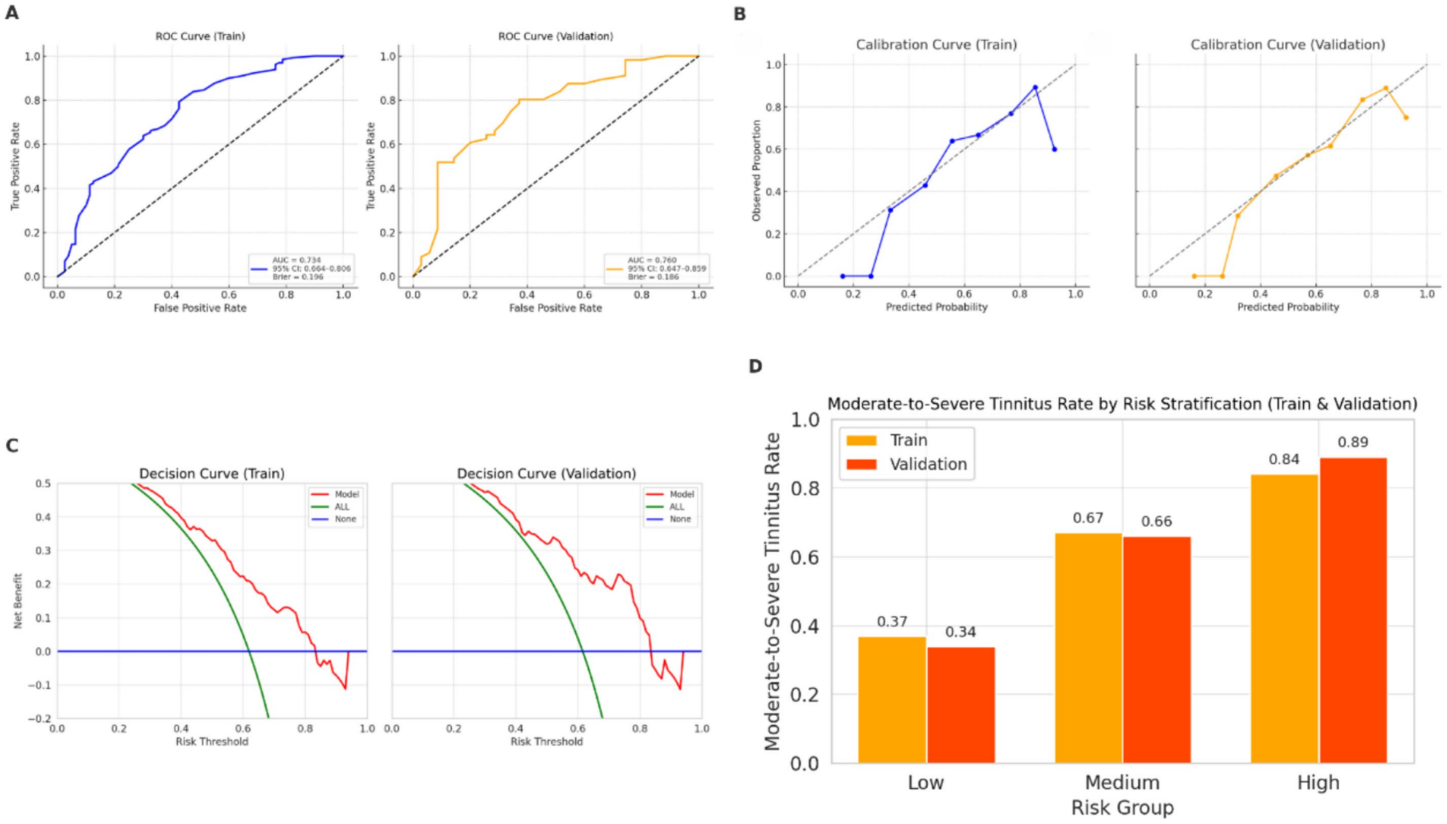
Performance evaluation of the prediction model for moderate-to-severe tinnitus. AUC, area under the receiver operating characteristic curve; OR, odds ratio; CI, confidence interval. **(A)** Receiver operating characteristic (ROC) curves for the training and validation sets, showing good discriminative ability in both sets. **(B)** Calibration curves based on 1,000 bootstrap resampling iterations, indicating good agreement between predicted probabilities and observed outcomes. Model calibration was further supported by the Hosmer–Lemeshow goodness-of-fit test. **(C)** Decision curve analysis (DCA) showing a positive net clinical benefit across a range of threshold probabilities in both sets. **(D)** Risk stratification based on tertiles of predicted probabilities in both the training and validation sets, showing a progressive increase in the observed incidence of moderate-to-severe tinnitus across low-, medium-, and high-risk groups.

The predictive model based on five independent variables—age, hearing loss severity, poor sleep quality, anxiety, and hypertension—demonstrated good discriminatory ability in both the training and validation sets. The area under the AUC was 0.734 (95% CI: 0.664–0.806) in the training set and 0.760 (95% CI: 0.647–0.859) in the validation set, demonstrating acceptable discriminative performance. The corresponding Brier scores were 0.196 and 0.186, respectively, suggesting low prediction error and good model accuracy. Calibration curves based on 1,000 bootstrap resampling iterations showed excellent agreement between predicted probabilities and observed outcomes. The Hosmer–Lemeshow goodness-of-fit test also supported adequate model fit in both the training (χ^2^ = 6.50, *p* = 0.591) and validation (χ^2^ = 7.38, *p* = 0.496) DCA further indicated that the model provided a positive net clinical benefit across a wide range of threshold probabilities in both sets, supporting its potential utility for individualized risk stratification. To assess the model’s stratification ability in practical settings, patients in the validation set were divided into three groups—low-, medium-, and high-risk—based on tertiles of predicted probabilities. The actual incidence of moderate-to-severe tinnitus increased progressively across these groups, confirming the model’s capacity to effectively stratify risk levels. These findings are comprehensively illustrated in Figure 1.

## 4 Discussion

Our multivariate model identified five independent predictors of moderate-to-severe tinnitus among hearing-impaired patients: older age, more severe hearing loss, comorbid sleep disturbance, higher anxiety levels, and hypertension. Age was significantly associated with tinnitus risk, likely reflecting age-related auditory degeneration and reduced neural plasticity (2, 4). Severe hearing loss also demonstrated a strong association with clinically significant tinnitus, consistent with the deafferentation theory (16, 26). Hypertension emerged as a modest yet independent predictor, echoing prior findings that cochlear microvascular dysfunction may exacerbate tinnitus susceptibility (27, 28). Most notably, sleep disturbance and anxiety were strong predictors, consistent with large cohort studies indicating elevated tinnitus odds among individuals with insomnia or anxiety disorders (29–31). In contrast, depression, despite its high comorbidity with tinnitus in univariate analysis, did not retain significance after multivariate adjustment—suggesting its effects may be mediated through sleep and anxiety.

These associations likely reflect multiple converging pathophysiological pathways. Cochlear damage from aging or noise exposure reduces afferent input, leading to maladaptive central gain and synchronized bursting in auditory cortex neurons—a mechanism supported by the stochastic resonance theory and top-down network reorganization (16, 26). Hypertension may contribute to cochlear ischemia, impaired ionic homeostasis, and oxidative stress, resulting in heightened neural excitability (27, 28). Sleep disturbance likely amplifies tinnitus via chronic hyperarousal: patients often exhibit elevated beta/gamma EEG activity during wake and sleep, diminished deep sleep, and HPA axis dysregulation with abnormal cortisol secretion (32, 33). Anxiety promotes hypervigilance and limbic overactivation (e.g., amygdala, insula), creating a feedback loop between tinnitus perception and emotional distress (31, 33, 34). Depression, though not independently predictive in our model, shares overlapping neurobiological substrates with tinnitus and may interact through common pathways, including HPA dysfunction and impaired coping (12, 35–37).

Our findings broadly align with existing epidemiological and neurobiological literature. Age and hearing loss are consistently identified as primary risk factors in large datasets such as NHANES and the UK Biobank (2, 4, 16). The strong link between poor sleep and tinnitus has been documented in multiple cross-sectional studies, including NHANES analyses, which report that insufficient or excessive sleep increases tinnitus risk independent of hearing status (29, 38). Similarly, anxiety has been repeatedly shown to double the odds of reporting bothersome tinnitus, and remains significant even after adjusting for hearing loss and other factors (30, 31). Although many studies report elevated depression rates in tinnitus sufferers (35, 36), our findings echo recent population-based analyses indicating that depression’s independent role is diminished once anxiety and sleep are controlled for (12, 30). The modest association between hypertension and tinnitus observed in our cohort is also supported by meta-analyses and large population studies such as the Tromsø Study (27, 28).

These findings have several implications for clinical practice. First, screening for sleep and anxiety disturbances should be standard in the management of tinnitus among hearing-impaired patients. Interventions such as cognitive-behavioral therapy (CBT) for insomnia or anxiety have shown efficacy in reducing tinnitus distress and improving quality of life (39, 40). Second, modifiable cardiovascular risk factors such as hypertension should be actively managed, as they may contribute to cochlear pathology. Third, treating underlying hearing loss with amplification or cochlear implants can improve both tinnitus perception and associated psychological distress (41). Finally, a multidisciplinary approach—integrating audiologic care, mental health support, and sleep interventions—may yield the best outcomes (42). Future research should explore causal directions through longitudinal designs, incorporate objective sleep and stress biomarkers, and examine genetic susceptibility to stress-mediated tinnitus (37). Understanding these pathways could inform preventive strategies and tailored interventions for high-risk subgroups.

### 4.1 Limitations

This study has several limitations. First, its cross-sectional design precludes any inference of causality; it is unclear whether poor sleep and anxiety precede tinnitus onset or result from it. Longitudinal studies are needed to clarify temporal relationships. Second, our depression measure may have been underpowered; structured diagnostic interviews or longer-term assessments might reveal more nuanced effects. Third, the study was conducted at a single tertiary care centre in western China, which may limit generalizability to other populations. Cultural, genetic, and environmental differences should be considered when interpreting these findings.

We also lacked objective audiometric and sleep data. Detailed hearing profiles (e.g., tinnitus pitch matching, residual inhibition tests) and objective sleep assessments such as polysomnography would enhance the accuracy of tinnitus phenotyping and allow better mechanistic insight. Biomarkers such as cortisol levels or heart rate variability could also clarify the role of neuroendocrine and autonomic dysregulation. Future research should incorporate multimodal approaches—including neuroimaging, genetics, and longitudinal follow-up—to build a more comprehensive model of tinnitus risk.

## 5 Conclusion

In conclusion, our study highlights the complex interplay between auditory and non-auditory factors in the development of moderate-to-severe tinnitus. While hearing loss and aging remain central predictors, the strong, independent roles of sleep disturbance and anxiety suggest that tinnitus should be considered a biopsychosocial condition. Interventions targeting these modifiable factors hold promise for reducing the incidence and severity of clinically significant tinnitus among hearing-impaired patients.

## Data Availability

The datasets presented in this article are not readily available because due to ethical and privacy protections, the raw data from this study cannot be shared publicly. This restriction is in compliance with the ethical approval granted by the Institutional Review Board of Qinghai University Affiliated Hospital (Approval No. P-SL-2024-056). Requests to access the datasets should be directed to CZ, 1403105471@qq.com.

## References

[1] Jarach CM Lugo A Scala M van den Brandt PA Cederroth CR Odone A. Global prevalence and incidence of tinnitus: a systematic review and meta-analysis. JAMA Neurol. (2022) 79:888–900. doi: 10.1001/jamaneurol.2022.2189, PMID: 35939312 PMC9361184

[2] Reisinger L Schmidt F Benz K Vignali L Roesch S Kronbichler M. Ageing as risk factor for tinnitus and its complex interplay with hearing loss-evidence from online and NHANES data. BMC Med. (2023) 21:283. doi: 10.1186/s12916-023-02998-1, PMID: 37533027 PMC10394883

[3] Chen Z Lu Y Chen C Lin S Xie T Luo X. Association between tinnitus and hearing impairment among older adults with age-related hearing loss: a multi-center cross-sectional study. Front Neurol. (2024) 15:1501561. doi: 10.3389/fneur.2024.1501561, PMID: 39741702 PMC11686227

[4] Oosterloo BC Croll PH Baatenburg de Jong RJ Ikram MK Goedegebure A. Prevalence of tinnitus in an aging population and its relation to age and hearing loss. Otolaryngol Head Neck Surg. (2021) 164:859–68. doi: 10.1177/0194599820957296 32988263 PMC8027937

[5] Clifford RE Maihofer AX Chatzinakos C Coleman JRI Daskalakis NP Gasperi M. Genetic architecture distinguishes tinnitus from hearing loss. Nat Commun. (2024) 15:614. doi: 10.1038/s41467-024-44842-x, PMID: 38242899 PMC10799010

[6] Chen NS Zhao YX Ma X Yu LS. Advances in the relevance of laryngopharyngeal reflux and tinnitus. Zhonghua Er Bi Yan Hou Tou Jing Wai Ke Za Zhi. (2019) 54:554–7. doi: 10.3760/cma.j.issn.1673-0860.2019.07.016, PMID: 31315368

[7] Maihoub S Mavrogeni P Molnár V Molnár A. Tinnitus and its comorbidities: a comprehensive analysis of their relationships. J Clin Med. (2025) 14:1285. doi: 10.3390/jcm14041285, PMID: 40004815 PMC11856243

[8] Molnár A Molnár V Mavrogeni P Maihoub S. The influence of carotid and vertebral Doppler ultrasonography and brain MRI abnormalities on hearing levels, tinnitus intensities and frequencies. Audiol Res. (2025) 15:29. doi: 10.3390/audiolres15020029, PMID: 40126277 PMC11932294

[9] Al-Lahham S Nazzal Z Massarweh A Saymeh D Al-Abed S Muhammad D. Prevalence and associated risk factors of tinnitus among adult Palestinians: a cross-sectional study. Sci Rep. (2022) 12:20617. doi: 10.1038/s41598-022-24015-w, PMID: 36450754 PMC9712604

[10] Biswas R Genitsaridi E Trpchevska N Lugo A Schlee W Cederroth CR. Low evidence for tinnitus risk factors: a systematic review and meta-analysis. J Assoc Res Otolaryngol. (2023) 24:81–94. doi: 10.1007/s10162-022-00874-y, PMID: 36380120 PMC9971395

[11] Meijers SM Rademaker M Meijers RL Stegeman I Smit AL. Correlation between chronic tinnitus distress and symptoms of depression: a systematic review. Front Neurol. (2022) 13:870433. doi: 10.3389/fneur.2022.870433, PMID: 35585851 PMC9108431

[12] Salazar JW Meisel K Smith ER Quiggle A McCoy DB Amans MR. Depression in patients with tinnitus: a systematic review. Otolaryngol Head Neck Surg. (2019) 161:28–35. doi: 10.1177/0194599819835178, PMID: 30909841 PMC7721477

[13] Oosterloo BC de Feijter M Croll PH Baatenburg de Jong RJ Luik AI Goedegebure A. Cross-sectional and longitudinal associations between tinnitus and mental health in a population-based sample of middle-aged and elderly persons. JAMA. Otolaryngol Head Neck Surg. (2021) 147:708–16. doi: 10.1001/jamaoto.2021.1049, PMID: 34110355 PMC8193541

[14] Goderie T van Wier MF Lissenberg-Witte BI Merkus P Smits C Leemans CR. Factors associated with the development of tinnitus and with the degree of annoyance caused by newly developed tinnitus. Ear Hear. (2022) 43:1807–15. doi: 10.1097/AUD.0000000000001250, PMID: 35729718 PMC9592178

[15] Rademaker MM Smit AL Stokroos RJ van Smeden M Stegeman I. Development and internal validation of a prediction model for the presence of tinnitus in a Dutch population-based cohort. Front Neurol. (2023) 14:1213687. doi: 10.3389/fneur.2023.1213687, PMID: 37602261 PMC10434772

[16] Hobeika L Fillingim M Tanguay-Sabourin C Roy M Londero A Samson S. Tinnitus risk factors and its evolution over time. Nat Commun. (2025) 16:4244. doi: 10.1038/s41467-025-59445-3, PMID: 40335454 PMC12059016

[17] Jafari Z Harari RE Hole G Kolb BE Mohajerani MH. Machine learning models can predict tinnitus and noise-induced hearing loss. Ear Hear. (2025). doi: 10.1097/AUD.0000000000001670, PMID: 40325514 PMC12352570

[18] Newman CW Jacobson GP Spitzer JB. Development of the tinnitus handicap inventory. Arch Otolaryngol Head Neck Surg. (1996) 122:143–8. doi: 10.1001/archotol.1996.01890140029007, PMID: 8630207

[19] McCombe A Baguley D Coles R McKenna L McKinney C Windle-Taylor P. Guidelines for the grading of tinnitus severity: the results of a working group commissioned by the British Association of Otolaryngologists, head and neck surgeons, 1999. Clin Otolaryngol Allied Sci. (2001) 26:388–93. doi: 10.1046/j.1365-2273.2001.00490.x, PMID: 11678946

[20] Nutrition and Metabolic Management Branch of China International Exchange and Promotive Association for Medical and Health Care Clinical Nutrition Branch of Chinese Nutrition Society Chinese Diabetes Society Chinese Society for Parenteral and Enteral Nutrition Chinese Clinical Nutritionist Center of Chinese Medical Doctor Association. Guidelines for medical nutrition treatment of overweight/obesity in China (2021). Asia Pac J Clin Nutr. (2022) 31:450–82. doi: 10.6133/apjcn.202209_31(3).0013, PMID: 36173217

[21] Mosli M Alkhathlan B Abumohssin A Merdad M Alherabi A Marglani O. Prevalence and clinical predictors of LPR among patients diagnosed with GERD according to the reflux symptom index questionnaire. Saudi J Gastroenterol. (2018) 24:236–41. doi: 10.4103/sjg.SJG_518_17, PMID: 29652032 PMC6080153

[22] Abraham ZS Kahinga AA. Utility of reflux finding score and reflux symptom index in diagnosis of laryngopharyngeal reflux disease. Laryngoscope Investig Otolaryngol. (2022) 7:785–9. doi: 10.1002/lio2.799, PMID: 35734054 PMC9194976

[23] Li H Jin D Qiao F Chen J Gong J. Relationship between the self-rating anxiety scale score and the success rate of 64-slice computed tomography coronary angiography. Int J Psychiatry Med. (2016) 51:47–55. doi: 10.1177/0091217415621265, PMID: 26681235

[24] Negeri ZF Levis B Sun Y He C Krishnan A Wu Y. Accuracy of the patient health questionnaire-9 for screening to detect major depression: updated systematic review and individual participant data meta-analysis. BMJ. (2021) 375:n2183. doi: 10.1136/bmj.n2183, PMID: 34610915 PMC8491108

[25] Zitser J Allen IE Falgàs N Le MM Neylan TC Kramer JH. Pittsburgh sleep quality index (PSQI) responses are modulated by total sleep time and wake after sleep onset in healthy older adults. PLoS One. (2022) 17:e0270095. doi: 10.1371/journal.pone.0270095, PMID: 35749529 PMC9232154

[26] Wang S Cha X Li F Li T Wang T Wang W. Associations between sleep disorders and anxiety in patients with tinnitus: a cross-sectional study. Front Psychol. (2022) 13:963148. doi: 10.3389/fpsyg.2022.963148, PMID: 35992459 PMC9389284

[27] Ausland JH Engdahl B Oftedal B Hopstock LA Johnsen M Krog NH. Tinnitus and cardiovascular disease: the population-based Tromsø study (2015-2016). BMJ Public Health. (2024) 2:e000621. doi: 10.1136/bmjph-2023-000621, PMID: 40018628 PMC11816207

[28] Figueiredo RR de Azevedo AA Penido NO. Tinnitus and arterial hypertension: a systematic review. Eur Arch Otorrinolaringol. (2015) 272:3089–94. doi: 10.1007/s00405-014-3277-y, PMID: 25190255

[29] Wang C Li S Shi M Qin Z Wang D Li W. Association between sleep and tinnitus in US adults: data from the NHANES (2007-2012). Medicine (Baltimore). (2024) 103:e40303. doi: 10.1097/MD.0000000000040303, PMID: 39470498 PMC11521005

[30] Hackenberg B Döge J O'Brien K Bohnert A Lackner KJ Beutel ME. Tinnitus and its relation to depression, anxiety, and stress-a population-based cohort study. J Clin Med. (2023) 12:1169. doi: 10.3390/jcm12031169, PMID: 36769823 PMC9917824

[31] Arsenault V Larouche J Désilets M Hudon M-A Hudon A. When the mind meets the ear: a scoping review on tinnitus and clinically measured psychiatric comorbidities. J Clin Med. (2025) 14:3785. doi: 10.3390/jcm14113785, PMID: 40507547 PMC12155966

[32] Bao X Feng X Huang H Li M Chen D Wang Z. Day-night hyperarousal in tinnitus patients. Sleep Med. (2025) 131:106519. doi: 10.1016/j.sleep.2025.106519, PMID: 40262425

[33] Patil JD Alrashid MA Eltabbakh A Fredericks S. The association between stress, emotional states, and tinnitus: a mini-review. Front Aging Neurosci. (2023) 15:1131979. doi: 10.3389/fnagi.2023.1131979, PMID: 37207076 PMC10188965

[34] Maihoub S Mavrogeni P Répássy GD Molnár A. Exploring how blood cell levels influence subjective tinnitus: a cross-sectional case-control study. Audiol Res. (2025) 15:72. doi: 10.3390/audiolres15030072, PMID: 40558411 PMC12190120

[35] Jiang Y Liu Q Ding Y Sun Y. Systematic review and meta-analysis of the correlation between tinnitus and mental health. Am J Otolaryngol. (2025) 46:104611. doi: 10.1016/j.amjoto.2025.104611, PMID: 40088765

[36] Lin X Liu Y Chen Z Wei Y Lin J Chen C. Association between depression and tinnitus in US adults: a nationally representative sample. Laryngoscope Investig Otolaryngol. (2023) 8:1365–75. doi: 10.1002/lio2.1134, PMID: 37899867 PMC10601571

[37] Bhatt IS Wilson N Dias R Torkamani A. A genome-wide association study of tinnitus reveals shared genetic links to neuropsychiatric disorders. Sci Rep. (2022) 12:22511. doi: 10.1038/s41598-022-26413-6, PMID: 36581688 PMC9800371

[38] Awad M Abdalla I Jara SM Huang TC Adams ME Choi JS. Association of sleep characteristics with tinnitus and hearing loss. OTO Open. (2024) 8:e117. doi: 10.1002/oto2.117, PMID: 38420352 PMC10900921

[39] Aazh H. Cognitive behavioural therapy (CBT) for managing tinnitus, hyperacusis, and misophonia: the 2025 Tonndorf lecture. Brain Sci. (2025) 15:526. doi: 10.3390/brainsci15050526, PMID: 40426697 PMC12109689

[40] W Beukes E Andersson G Fagelson M Manchaiah V. Internet-based audiologist-guided cognitive behavioral therapy for tinnitus: randomized controlled trial. J Med Internet Res. (2022) 24:e27584. doi: 10.2196/27584 35156936 PMC8887633

[41] Olze H Szczepek AJ Haupt H Förster U Zirke N Gräbel S. Cochlear implantation has a positive influence on quality of life, tinnitus, and psychological comorbidity. Laryngoscope. (2011) 121:2220–7. doi: 10.1002/lary.22145, PMID: 21898434

[42] Lee HY Jung DJ. Recent updates on tinnitus management. J Audiol Otol. (2023) 27:181–92. doi: 10.7874/jao.2023.00416, PMID: 37872753 PMC10603282

