# Supplementary figures and images for: Identifying psychological and clinical risk factors for moderate-to-severe tinnitus in older patients with hearing loss: a multivariable prediction model
Source: Front Neurol. 2025 Jul 23;16:1647071. doi: 10.3389/fneur.2025.1647071 (PMC12325010; doi:10.3389/fneur.2025.1647071)

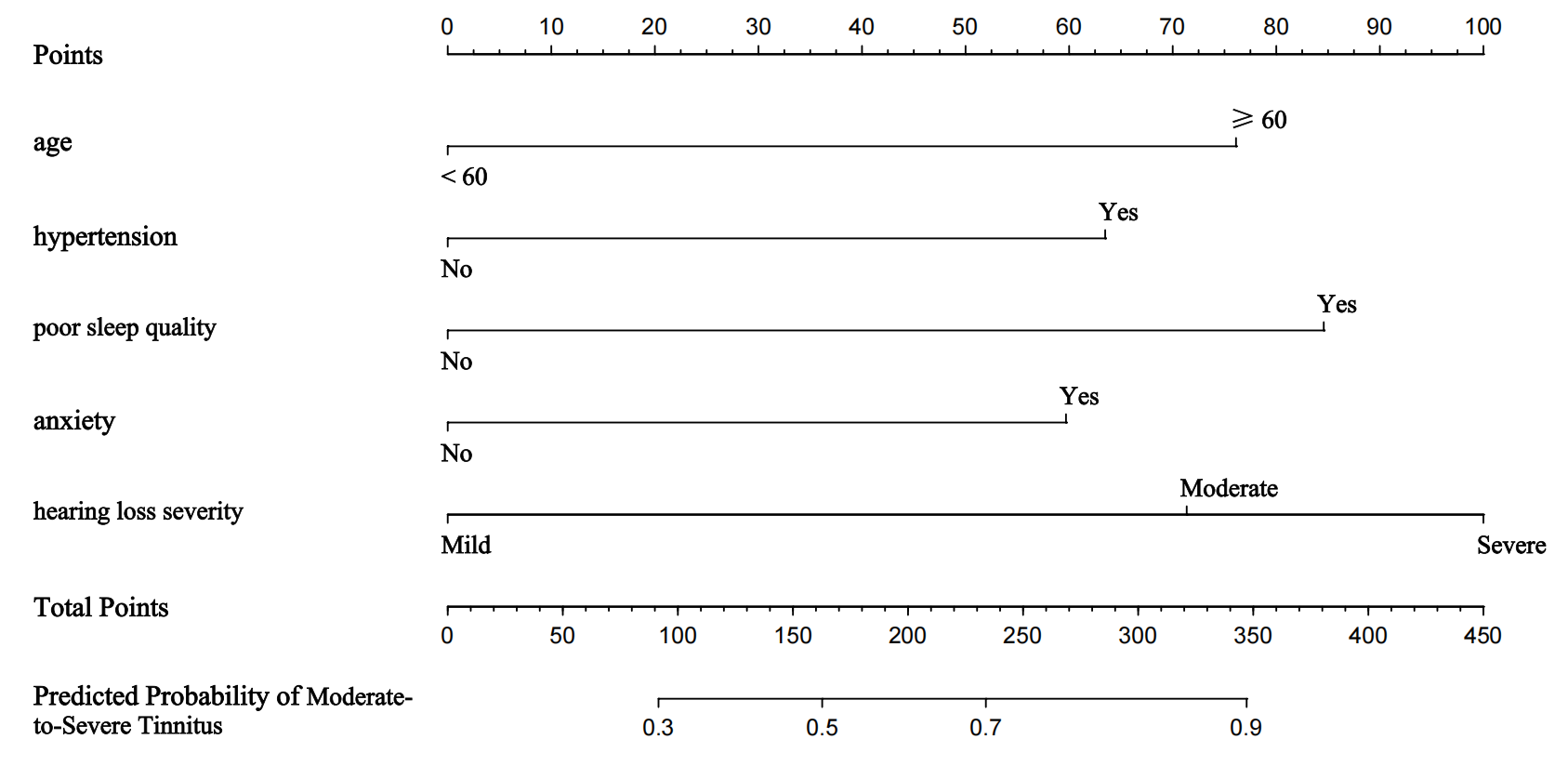

Supplement: Supplementary file 1 [file Image_1.PNG]
